# Prediction of congenital hypothyroidism based on initial screening thyroid-stimulating-hormone

**DOI:** 10.1186/s12887-016-0559-0

**Published:** 2016-02-02

**Authors:** David S. Saleh, Sarah Lawrence, Michael T. Geraghty, Patricia H. Gallego, Karen McAssey, Diane K. Wherrett, Pranesh Chakraborty

**Affiliations:** Division of Pediatric Endocrinology and Metabolism, Department of Pediatrics, University of Ottawa, Ottawa, ON Canada; Department of Pediatrics, University of Ottawa, Ottawa, ON Canada; Department of Pediatrics, Queen’s University, Kingston, ON Canada; Department of Pediatrics, University of Western Ontario, London, ON Canada; Division of Pediatric Endocrinology, Department of Pediatrics, McMaster University, Hamilton, ON Canada; Division of Endocrinology, Department of Pediatrics, Hospital for Sick Children, University of Toronto, Toronto, ON Canada; Newborn Screening Ontario, Children’s Hospital of Eastern Ontario, Ottawa, Canada

**Keywords:** Congenital hypothyroidism, Thyroid stimulating hormone, Thyroid hormone, Newborn screening

## Abstract

**Background:**

In thyroid-stimulating-hormone (TSH)-based newborn congenital hypothyroidism (CH) screening programs, the optimal screening-TSH cutoff level is critical to ensuring that true cases of CH are not missed. Screening-TSH results can also be used to predict the likelihood of CH and guide appropriate clinical management. The purpose of this study is to evaluate the predictive value of various screening-TSH levels in predicting a diagnosis of CH in the Ontario Newborn Screening Program (ONSP).

**Methods:**

The initial screening and follow-up data of 444,744 full term infants born in Ontario, Canada from April 1, 2006 to March 31, 2010 were analyzed. Confirmed CH cases were based on local endocrinologists’ report and initiation of thyroxine treatment.

**Results:**

There were a total of 541 positive screening tests (~1/822 live births) of which 296 were true positives (~1:1,500 live births). Subjects were further subdivided based on screening-TSH and positive predictive values (PPV) were calculated. Twenty four percent in the 17–19.9 mIU/L range were true positives. In the 17–30 mIU/L range, 29 % were true positives with a significantly higher PPV for those sampled after (43 %) rather than before (25 %) 28 h of age (*p* < 0.02). Seventy three percent of neonates with an initial screening-TSH of ≥ 30 mIU/L and 97 % of those with ≥ 40 mIU/L were later confirmed to have CH.

**Conclusions:**

Infants with modestly elevated screening positive TSH levels between 17 and 19.9 mIU/L have a significant risk (24 %) of having CH. The very high frequency of true positives in term newborns with initial TSH values ≥ 30mIU/L suggests that this group should be referred directly to a pediatric endocrinologist in an effort to expedite further assessment and treatment. Screen positives with a modestly elevated TSH values (17-19.9 mIU/L) need to be examined in more detail with extended follow-up data to determine if they have transient or permanent CH.

## Background

Thyroid hormone is essential for normal central nervous system development – especially in the first 3 years of life [1]. For children with untreated CH, the result is permanent, irreversible cognitive delay, impaired motor function and growth. This can be prevented by early detection and treatment [2, 3]. As a result, screening programs to detect CH in the neonatal period were developed in the early 1970’s and adopted by many countries throughout the world. In Ontario, Canada, a primary TSH with no second-tier test strategy has been used by Newborn Screening Ontario (NSO) since April 2006.

Since the introduction of screening programs, screening-TSH cutoffs and the predictive value of various screening-TSH levels has been the subject of debate [4–6], and a variety of strategies are used. Some programs choose a standard TSH screen cutoff, often in the 20–30 mIU/L range, and consider all newborns below the cutoff as negative for CH. Others choose 2 cutoffs: a standard cutoff, and a second lower threshold, as low as 6 mIU/L. This second group is considered “low-risk” for CH, and goes on to have follow up thyroid function testing [4, 7]. In such programs screening samples are typically collected at a later age, which also explains the lower threshold in that we know that TSH levels decline with time after birth. Regardless of the strategy employed, there has long been established a correlation between the initial screening-TSH level and the risk of CH [8]. The primary purpose of this study is to formally evaluate the predictive value of the initial screening-TSH result in Ontario.

## Methods

Subjects were identified from all newborns screened in Ontario, Canada between April 1, 2006 and March 31, 2010. Confirmatory thyroid function testing bloodwork, thyroid scan data and patient management information was collected by the individual centre and submitted to the ONSP. We collected all data directly from the ONSP representing the 5 major pediatric centers in Ontario. Aggregate NSO information is made publically available in an annual report posted on the NSO website (www.newbornscreening.on.ca). The record level data used for this analysis is not publically available. Inquiries about access to NSO data should be directed to Dr. Pranesh Chakraborty (Executive Director, NSO).

Stop date was chosen as a new data collection system was implemented beyond this date. Inclusion criteria included term newborns (≥37 weeks gestational age) whose screening blood sample was collected later than 24 h of age. All newborns screened <24 h are routinely re-screened with a bloodspot sample as part of NSO protocol. Subjects of mothers who were reported to be on anti-thyroid medication were excluded. Multiple birth newborns were not excluded. Screening blood samples were collected by heel stick bloodspot on filter paper, and analyzed using an automated Perkin-Elmer autoDELFIA immunoassay measuring TSH in mIU/L blood volume. No changes to the assay occurred during the study period. Newborns with an initial screening-TSH ≥17 mIU/L had confirmatory duplicate TSH bloodspot testing on the same sample and were referred to a local physician for further evaluation and management if the average of three TSH results was ≥ 17 mIU/L. Data collected consisted of the short term follow up information routinely collected by NSO, including screening and confirmatory TSH values, time of screening sample collection, gestational age, birth-weight and final diagnosis (based on local physician’s report). Subjects were defined as having true CH if they were started on thyroxine replacement therapy at the discretion of their local endocrinologist – a decision based primarily on confirmatory plasma TSH and fT4 testing, and thyroid imaging (thyroid scan or ultrasound).

The Fisher Exact Test was used to compare positive predictive values between age-of-sample collection groups <28 and ≥ 28 h (median age of sample collection was 28 h).

Patients in the study were anonymized and assigned a unique study identifier number. This study was approved by the CHEO Research Ethics Board.

## Results

Subjects included 444,744 term newborns born between April 1, 2006 and March 31, 2010, of whom 541 newborns screened positive representing a screen positive rate of approximately 1 in 822 newborns. Follow-up data was available for all screen positive subjects, and 296 were later confirmed to have CH representing an incidence of approximately 1:1,500 term newborns.

Table 1 shows the positive predictive value and sensitivity for various TSH screen cutoff levels. At a screening-TSH cutoff of 17 mIU/L, 296 of 541 newborns were diagnosed with CH and started on treatment yielding a positive predictive value of 54.7 %. For the purposes of this analysis, we made the assumption that none of the 444,193 newborns who screened negative actually had CH, yielding a sensitivity for the program of close to 100 %. When the TSH screening cutoff is increased to 20 mIU/L, although the positive predictive value increases to 72.9 %, the sensitivity decreases significantly to 83.8 %.

**Table 1:** Positive predictive values and sensitivities for the CH ONSP at various screening-TSH cutoff values

TSH Screen Cutoff (mIU/L)	True Positives/ Screen Positives	Positive Predictive Value (%)	Sensitivity (%)
≥17	296/541	55 %	~100 %
≥20	248/340	73 %	84 %
≥30	198/211	94 %	67 %
≥40	172/177	97 %	58 %
≥50	157/159	99 %	53 %

Follow-up data from the 541 screen-positive infants was then analyzed based on various screening-TSH ranges (Table 2). Of the 201 newborns with screening-TSH results between 17–19.9 mIU/L, 48 were later confirmed to have CH, yielding a positive predictive value (PPV) of 23.9 % for those who screen positive in this range. The PPV increases to 39 % for screening-TSH results of 20–29.9 mIU/L, 76.5 % for a screening-TSH of 30–39.9 mIU/L, and 97.2 % for a screening-TSH ≥ 40.0 mIU/L.

**Table 2:** Positive predictive values for the CH ONSP at various screening-TSH ranges

TSH Screen Range (mIU/L)	Screen Positives	True Positives	False Positives	Positive Predictive Value
17–19.9	201	48	153	24 %
20–29.9	129	50	79	39 %
30–39.9	34	26	8	76 %
≥40	177	172	5	97 %

Newborns with ‘grey zone’ screening TSH of 17–29.9 mIU/L, the PPV was 30 % (98 of 330). Of confirmed positive cases with screening TSH 17-29.9 mIU/L, 47 % (20 of 43) had a low free T4 on confirmatory thyroid function testing (according to local lab normal ranges), compared with 66 % (63 of 96) of those whose screening TSH was ≥30. Free T4 data was only available for 47 % of cases. Of those subjects whose screening-TSH level was 17–29.9 mIU/L, 27 % (12 of 45) had evidence of thyroid dysgenesis on thyroid scan, versus 68 % (82 of 120) in the screening-TSH ≥30 mIU/L range (Table 3). Thyroid scan data was only available for 56 % (165 of 294) of confirmed positives overall [46 % (45 of 98) in the 17–29.9 mIU/L range, and 61 % (120 of 196) in the screening-TSH ≥30 mIU/L range]. Although confirmatory serum TSH levels were likely drawn for all screen positive patients as per standard clinical practice, we received data only 169/296 (57 %) of subjects. In 138 of 296 cases, we received confirmatory TSH, fT4 and thyroid scan data.

**Table 3:** Thyroid scan results for confirmed CH cases

Thyroid Scan result	TSH 17–30 group (frequency [%])	TSH >30 group (frequency [%])	All Confirmed Positive cases
Normal Scan	28 [62]	33 [28]	61 [37]
Ectopic gland	8 [18]	53 [44]	61 [37]
Athyrosis	1 [2]	29 [24]	30 [18]
Other Dysplasia	3 [7]	0 [0]	2 [1]
All Dysplasia	12 [27]	82 [68]	94 [57]
Decreased uptake	5 [11]	5 [4]	10 [6]
Total	45	120	165

**Fig. 1:**
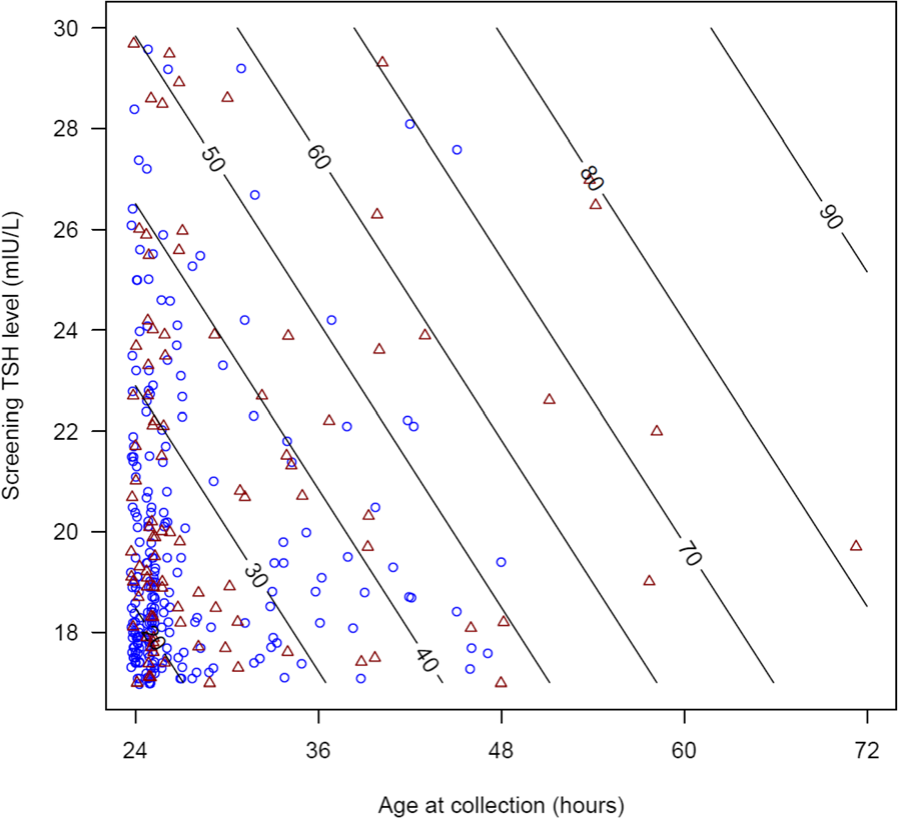
Scatter plot of screening-TSH level 17–30 mIU/L and age of sample collection. Red triangles represent true positive subjects and blue circles represent false positive subjects whose screening TSH level was 17–30 mIU/L. Contours represent estimate of probability of CH for any given screening-TSH/age of sample collection pair

The PPV of newborns with screening-TSH levels between 17–30 mIU/L and whose blood sample was collected at >28 h of age was significantly higher than those whose samples were collected 24-28 h of age (43 % vs. 25 %; *p* = 0.002; Fig. 1).

## Discussion

This cohort of 444,744 newborns yielding 541 screen positive subjects represents one of the largest recently published analyses of a CH screening program. The incidence of CH of 1:1,500 is comparable to reports in other jurisdictions, although higher than most industrialized, iodine replete countries and suggests efficient ascertainment of mild cases of CH [9]. Diagnosed newborns include those with both permanent and transient forms of CH. This could lead to over-diagnosis of CH as many clinicians will treat confirmed screen positive patients until 3 years of age, then reassess to determine if they have a transient form of CH [10]. Elevated screening and diagnostic TSH values can also be secondary to severe illness [11], maternal TSH receptor blocking antibodies [12], maternal iodine deficiency [13, 14] and excess [15], infant exposure to iodine [16], errors in the screening procedure, and newborns conceived by in vitro fertilization [17] or delivered by caesarian section [18].

There is a wide range of screening-TSH cutoffs used in neonatal screening programs including a low of 6 mIU/L in Wales [4], to a high of 30 mIU/L in Turkey [19]. Much of this discrepancy has to do with the age of sample collection (as TSH falls over the first few days of life), or specific assay used to measure TSH. Hence, our program’s data based on a TSH cutoff of 17 mIU/L may not be generalizable to other programs that collect bloodspot samples at different ages, or use a different TSH immunoassay. Some programs use a TSH screen cutoff of 20 mIU/L. Increasing the cutoff in Ontario from 17 to 20 mIU/L would result in an unacceptable decrease in sensitivity from 100 % to 84 %, thereby missing 16 % of currently identified cases. Of the 20 patients with a screening TSH level in the 17–20 range, at least one had athyreosis indicated by thyroid scan (thyroglobulin level was not measured), and 9 had a low free T4 values on confirmatory thyroid function testing, suggesting classical CH. This underscores the importance of maintaining a low screening cutoff level of at most 17 mIU/L in order to detect an acceptable percentage of those with CH.

The positive predictive value of newborns with mild to moderate elevations of screening-TSH values in the 17–30 range is 30 %. This increases significantly to 76 % with a screening-TSH of 30-39.9 mIU/L, and to 97 % with a screening-TSH ≥40 mIU/L. This is consistent with a published report demonstrating that 41 % of newborns with TSH levels between 20–50 mIU/L had CH [8]. Given the high PPV of 94 % for true CH with a screening TSH over 30 mIU/L and the serious, permanent developmental consequences of untreated CH, we propose that all such newborns should be referred directly for specialist evaluation in an effort to expedite further assessment and treatment.

Time of sample collection is important to consider in conjunction with absolute screening-TSH value in predicting the likelihood of CH. When examining subjects with mild to moderately elevated screening-TSH levels of 17–30 mIU/L, the PPV in newborns whose sample was collected ≥28 h of age was statistically higher than those whose samples were collected at 24–28 h of age (43 % vs. 25 %, *p* = 0.002). TSH-based screening programs could potentially use age of sample collection data, in addition to absolute screening TSH level, as a better tool for capturing true positive cases and predicting the risk of true CH (Fig. 1).

Limitations of this study include our assumption that no infants with a screening-TSH result <17 mIU/L actually had CH, an assertion that may not be accurate. There is no formal system in place that captures CH patients who screen negative; however there were no reports of false negative screening results in children with clinically identified CH during the study period. More recently, there was one screen-negative infant confirmed to have CH in the context of a strong family history of hypothyroidism. This patient had a mild form of CH. We would anticipate that most false screen-negative patients would be brought to the attention of the program or participating pediatric endocrinologist, particularly those patients with permanent CH and significant developmental morbidity secondary to untreated CH.

The diagnosis of CH was based on local endocrinologist report and initiation of thyroxine treatment. This raises the limitations related to individual variations in clinical practice as well as determination of transient versus permanent CH. A Japanese study that found a positive correlation between the prevalence of CH and regions with a lower concentration of adult and pediatric endocrinologists [20]. In Ontario, although non-specialists physicians are occasionally involved in the management of CH patients, the decision to treat is typically made by - or in conjunction with - a pediatric endocrinologist in virtually all cases. This may have reduced but would not eliminate the likelihood of overdiagnosis and treatment of some screen positive children.

A proportion of subjects may have a transient or mild form of CH, particularly those whose screening-TSH level was in the lower end of the range. This notion is supported by Kemper et al. who found that 38 % of children labeled as having CH by NBS no longer received thyroxine by age 4 [21]. Treatment of such infants is becoming increasingly controversial [6], as the neurodevelopmental consequences of withholding treatment in such cases is unclear. Despite this, in our study, of those subjects whose screening-TSH level was 17–29.9 mIU/L, approximately 1 in 4 had evidence of thyroid dysgenesis on thyroid scan, and nearly half had a low free T4 on confirmatory thyroid function testing, suggesting the possibility of clinically significant CH. Thyroid scan and free T4 data was not available for all subjects. In addition, all confirmatory TSH values reported to the ONSP in confirmed cases (representing 57 % of cases) were elevated above the reference range of the local laboratory.

Further clinical and biochemical evaluation of confirmed cases 3 years post-diagnosis will be necessary to conclusively determine the true incidence of permanent CH. Unfortunately, this study was not designed to collect such information. Even complete imaging data would be insufficient, as some forms permanent thyroid dyshormonogenesis have a normal thyroid imaging at diagnosis [22]. Although treatment of mild or transient cases of CH is controversial, in the absence of a clear way to distinguish transient and permanent CH in the newborn period, and of data to support conservative observation in those with transient CH, treatment is indicated.

## Conclusions

This study demonstrates that infants with modestly elevated screening-TSH values between 17–19.9 mIU/L have a significant risk (24 %) of having CH. The high frequency of true positives in those with screening-TSH levels over 30 mIU/L suggests that this group should be directly referred for specialist evaluation. Samples collected after 28 h of age have a significantly higher likelihood of being true positives with modest screening TSH elevations in the 17–30 mIU/L range – a finding that has possible implications in improving sensitivity and accuracy of identifying CH cases. Those who screen positive with TSH values between 17–20 mIU/L require further analysis and long-term follow up to determine if they have transient versus permanent CH.

## Abbreviations

TSH: Thyroid stimulating hormone
CH: Congenital hypothyroidism
ONSP: Ontario newborn screening program
FT4: Free thyroxine
PPV: Positive predictive value
NSO: Newborn screening Ontario
